# Regression-based normative data for social health scale for the elderly (short version) in eastern China

**DOI:** 10.1186/s12955-020-01306-2

**Published:** 2020-03-04

**Authors:** Zhe-Bin Yu, Cheng-Zhen Bao, Meng-Yin Wu, Dan-Jie Jiang, Xiao-Cong Zhang, Shu-Juan Lin, Ming-Juan Jin, Jian-Bing Wang, Meng-Ling Tang, Kun Chen

**Affiliations:** 1 Division of Epidemiology and Health Statistics, Department of Public Health, Zhejiang University School of Medicine, Hangzhou, 310058 Zhejiang China; 2 Beijing Obstetrics and Gynecology Hospital, Capital Medical University, Beijing, 100006 China; 3 Cancer Institute/Department of Epidemiology and Health Statistics, The second affiliated hospital, Zhejiang University School of Medicine, 866 Yuhangtang Road, Hangzhou, 310058 China

**Keywords:** Social health, Social support, Elderly, Normative data, Regression-based norm

## Abstract

**Backgrounds:**

Social Health Scale for the Elderly short version (SHSE-S) is a psychometrically sound instrument that comprehensively assesses the social health status of older adults in China. The aim of the present study was to establish continuous normative data of SHSE-S.

**Methods:**

We conducted a multicenter cross-sectional study among 31 communities in eastern China. Older adults aged 60 years and above were invited to participate in the study. Each participant was interviewed in-person to finish a structured questionnaire. The SHES-S score was calculated and standardized for each participant. We split the sample into generation and validation datasets and compared the distribution of SHSE-S score between two datasets. Multivariable linear regression was used to assess the SHSE-S score and demographic variables. Regression-based norms were built using a four-step process.

**Results:**

A total of 6089 participants (51.2% females) aged 60 years old and above (mean age = 71.3, SD = 8.0) were enrolled as the normative sample. No significant difference was found between the distribution of SHSE-S standardized score in the generation (*N* = 2392) and validation (*N* = 3697) datasets. Multivariable linear regression showed that females, higher education levels were positive indicators while aging, living alone, divorced or never married, multimorbidity were negative factors. The regression-based norm which taking demographic factors into account was established and a user-friendly worksheet was also provided to facilitate the scoring and norming of the SHSE-S.

**Conclusions:**

The population-based regression norm of SHSE-S can be a useful tool for assessing the social health status of the Chinese elderly population.

## Introduction

Health has long been considered as not just the absence of disease but also the presence of physical, psychological and social well-being [1]. A study redefined the comprehensive health in US older adults found that certain diseases and health behaviors were far less important than mental health and social health [2]. Unlike physical health [3–5] and mental health [6, 7], instruments which specially designed for evaluating the social health status of the aged population in China is still scarce.

The definition of social health contains both individual-level social health and the social health of society or a population [8]. Currently, scales relating to social health including RAND Social Health Battery, Social Dysfunction Rating Scale, Structured and Scaled Interview to Assess Maladjustment are not suitable for Chinese elderly population mainly due to the cultural heterogeneity and lack of assessment on the society level of the social health. Previously we have developed the first scale which combined both individual and society level of social health named “Social Health Scale for the Elderly (SHSE)” to evaluate the social health status for the elderly population in China. The development process had been described elsewhere [9]. There are 2 versions of this scale (long version contains 25items while the short version contains 14 items). The short version is more convenient to use and provides reliable information at the same time. Normative data can be really important to help interpret the result of a certain test [10]. Bao et al. previously built a percentile norm based on cross-sectional data of 2396 older adults in Hangzhou. However, this norm has not been validated in other populations in Zhejiang Province yet. Recently, an alternative approach to derive continuous norms using multivariate regression models have been proposed [11] and widely used in neuropsychological and social related measures [12–14]. This regression-based approach is useful insofar as it allows an individual’s predicted score on a measure to reflect specific demographic characteristics [15]. Using such an approach could reduce biases due to different characteristics derived from an unknown population. With the normative data generated in this study, the SHSE scale could hopefully serve as an effective tool to assess the social health status of elderly population in China. Those elderly with vulnerable social health status can be identified by community clinicians after finish this scale by comparing their score to the normative data. Further targeted intervention strategies can be made to improve the comprehensive health status for older adults. In this study, we used cross-sectional data among 6089 community-dwelling participants aged 60 years old and above to build normative data of SHSE-S for the elderly population in Zhejiang. We aimed to 1) Verify the distribution of SHSE-S scores in Zhejiang Population; 2) use a regression-based approach to build normative data and reduce confounding variables such as age, gender, educational level, etc.

## Methods and materials

### Study population

A multicenter cross-sectional study among the community-dwelling elderly was conducted in 17 communities of 2 districts in Hangzhou (8 in Gongshu and 9 in Xihu) to testify the reliability and validity of SHSE. The details have been described elsewhere [9]. We further conducted a cross-sectional study following the same process in 14 communities in Zhejiang Province (8 in Hangzhou, 4 in Jinhua, 2 in Huzhou) from Jan 1st, 2018 to Sep 30th, 2018. The inclusion criteria were aged 60 years or older and had been living in the communities for at least five years. Our exclusion criteria were as follows: 1. People who were bed-ridden or can’t take care of themselves due to serious physiological or psychological illness; 2. People who had vision or hearing disorders which can’t accomplish the interview. 3. Participants with dementia or reported difficulty in finishing the scale during the interview. After checking the Electronic Health Records in the community health services center, older adults who met the exclusion criteria were excluded. The total sample size of participants for each community was determined priory while sample size in each sex and age group strata was calculated based on the local population structure. Participants were conveniently selected by the general practitioner at the local community health service center, followed by the interviewers to complete the questionnaire. The Health Commission of Zhejiang Province ensures that every inhabitant is registered at a general practitioner and up to now the cover rate was 82.04% for older adults [16], which means the source of the participants is representative of the population. Written informed consent was obtained from all participants after being told the purpose and content of this study. We conducted face-to-face interviews for every participant by a well-trained interviewer (medical students or medical stuff) to finish a structured questionnaire at the local community health service center. Questionnaire completed by proxy or participants themselves were forbidden in the current study. The study protocol was approved by the Ethics committee at Zhejiang University, School of Medicine.

### Assessment of social health status

The developing process of the SHES-S has been described elsewhere. There is a total of 3 dimensions and 14 items in this scale. Item 1–4 (1. Being supported in the major decision; 2. Being emotionally cared for; 3. Being comforted; 4. Being helped with daily chores during illness) belong to dimension Social Support (SS), item 5–8 (5. Participating in a collective recreational activity; 6. Communication with children; 7. Communication with friends; 8. Interests and hobbies) belong to dimension Social Adjustment (SA) while item 9–14 (9. Manufactured landscape; 10. Public transit facility; 11. Fitness/recreation facility; 12. Medical institution; 13. Organizing activity; 14. Free public service) belongs to the dimension of Perceived Environment Resources (PER). The raw score calculation process was listed in Supplemental Table 1.

The raw score of each dimension was calculated by summing the raw score of the corresponding items. Generally, a higher score indicates a better status of social health. We further transformed the raw scale scores to 0–100 scale (standardized score) using the same way as the SF-36 health survey [17].

$$ \mathrm{Transformed}\ \mathrm{Scale}=\left(\mathrm{Actual}\ \mathrm{raw}\ \mathrm{score}-\mathrm{lowest}\ \mathrm{possible}\ \mathrm{raw}\ \mathrm{score}\right)/\left(\mathrm{Possible}\ \mathrm{raw}\ \mathrm{score}\ \mathrm{range}\right)\ast 100 $$

Formulas for the scoring standard score was listed in Supplemental Table 2. This transformation converts the lowest and highest scores to zero and 100, respectively. Standardized scores between these values represent the percentage of the total possible score achieved.

### Covariates

Demographic information including gender, calendar age, current living arrangement, current married status, education levels were also collected. The region of the local community where participants lived in were coded into urban or rural according to the national statistical zoning and urban-rural division code (http://www.stats.gov.cn/tjsj/tjbz/tjyqhdmhcxhfdm/2018/index.html). Comorbidities including the prevalence of 27 common chronic diseases were determined based on the participants’ self-report to the question, “Do you suffer, or were you told by a doctor that you suffer from the following problems in the past one year?” [18, 19] Chronic disease included hypertension, ischemic heart disease, cerebrovascular disease, diabetes, chronic obstructive pulmonary disease, Parkinson’s disease, arthritis, osteoarthritis, etc. The number of prevalent chronic diseases for each participant was calculated and categorized into none disease, 1 kind of disease, 2 kinds of diseases, 3 or more than 3 kinds of diseases.

### Statistical analysis

The raw scores of SHES-S, three dimensions and each item were presented as mean ± standard deviation. Data collected in 2017 (Xihu, Gongshu) were used as generation dataset while collected in 2018 (Anji, Jianggan, Yiwu) were used as validation dataset. We first compared the distribution of SHSE-S scores in the validation dataset with the percentile norm built in generation dataset. Participants in validation dataset were categorized into deciles according to the established norm in training set. Then we compared the distribution between validation dataset and generation dataset using Chi-square goodness of fit test.

Age was centered (calendar age-70) before performing multivariate regression. The effects of demographic variables on the SHSE-S scores and three dimensions’ scores were evaluated by regressing the raw scores on gender, age, age^2^, education, education^2^, married status, living arrangement, region and comorbidity. Gender was coded as male = 1, female = 2. Education was combined into four levels: 1 = illiterate, 2 = primary school, 3 = middle school, 4 = high school or above (modeled as continuous variables to allow the inclusion of polynomial terms for education). Married status was dummy coded into three groups (married, widowed, divorced or never married or others) with married as the reference group. Living arrangement was dichotomized coded into living alone or not (living alone = 1, living with others = 2). The region was coded as urban = 1, rural = 2. Comorbidity was dummy coded with 4 categories: 0, 1, 2, 3 or above with 0 as a reference. All two-way interactions were also included in the model. Non-significant predictors were excluded from the full models but no predictor was removed as long as it was also included in a higher order term or interaction term in the model [20]. Bonferroni method was used for the correction of the significance level. The assumptions of regression analysis were tested for all the models. Homoscedasticity was evaluated by grouping the participants into quartiles of the predicted scores and applying the Levene test. The existence of multicollinearity was detected by calculating VIFs (< 10 considered no multicollinearity). Potential influential cases were identified by calculating Cook’s distance. The normality of the residuals was investigated by conducting Kolmogorov-Smirnov tests on the standardized residuals.

Regression based norming was performed in a four-step process [21]. First, predicted scores of SHSE-S were calculated for each participant using the multivariate regression model. Second, residuals were calculated (residuals = observed SHSE-S score – predicted SHSE-S score). Altman-Bland method was used to evaluate the consistency between observed score and predicted score [22]. Third, the residuals were standardized according to the standard deviation of the residuals in the study sample. Fourth, the percentile of standardized residuals was calculated using standard normal cumulative distribution function (if the assumption of normality was met) or empirical cumulative distribution function. Normative data of three dimensions (social support, social adjustment and perceived environment resources) were also calculated following the same procedure.

All analyses were performed within the R software 3.4.1. Altman-Bland method was conducted using R package “MethComp”. A two-tailed *P* value < 0.05 was considered statistically significant.

## Results

### Distribution of demographic characteristics of the study population

The distribution of demographic characteristics between generation and validation datasets were presented in Table 1. A total of 2392 participants were included in the generation dataset and 3697 were included as validation dataset. There were 1226 females (51.25%) in the generation dataset and 1891 (51.15%) in the validation dataset. The mean age (71.96 ± 8.01) in validation dataset was higher than that of the generation dataset (70.66 ± 8.01). Participants in validation dataset had a significantly lower education level (high school or above 19.45% vs 39.67%, *P* < 0.001), a higher proportion of living in rural area (60.48% vs 53.51%, *P* < 0.001) and a higher proportion of having 3 or more than 3 chronic diseases (23.88% vs 17.77%, *P* < 0.001) comparing to the generation dataset.

**Table 1:** Distribution of demographic factors and standardized score of Social Health Scale for the Elderly short version (SHSE-S) in generation and validation dataset

Demographic factors	Generation dataset (*N* = 2392)	Validation dataset (*N* = 3697)	*P* value
Gender, n (%)			0.957
Male	1166 (48.75)	1806 (48.85)	
Female	1226 (51.25)	1891 (51.15)	
Age group, n (%)			< 0.001
60~69	1210 (50.59)	1657 (44.82)	
70~79	812 (33.95)	1307 (35.35)	
≥80	370 (15.46)	733 (19.83)	
Education, n (%)			< 0.001
Illiterate	542 (22.66)	734 (19.85)	
Primary school	411 (17.18)	1212 (32.78)	
Middle school	487 (20.36)	1012 (27.37)	
High school or above	949 (39.67)	719 (19.45)	
Married status, n (%)			< 0.001
Married	1636 (68.39)	2867 (77.55)	
Widowed	437 (18.27)	726 (19.64)	
Divorced, never married or other	319 (13.34)	104 (2.81)	
Region, n (%)			< 0.001
Urban	1112 (46.49)	1461 (36.52)	
Rural	1280 (53.51)	2236 (60.48)	
Living arrangement, n (%)			0.091
Living alone	230 (9.62)	407 (11.01)	
Living with others	2162 (90.38)	3290 (88.99)	
Comorbidity, n (%)			< 0.001
0	604 (25.25)	867 (23.45)	
1	790 (33.03)	1254 (33.92)	
2	573 (23.95)	693 (18.74)	
3 or above	425 (17.77)	883 (23.88)	
Standardized score of social health, mean ± SD			
SHSE-S	42.80 ± 13.00	40.13 ± 12.27	< 0.001
Social Support	31.63 ± 19.19	29.29 ± 14.35	< 0.001
Social Adjustment	38.07 ± 20.44	37.78 ± 22.58	< 0.001
Perceived Environment Resources	58.70 ± 19.91	53.32 ± 18.30	< 0.001

*P* values were derived using Chi-square test for categorical variables and t-test for continuous variables between generation and validation datasets

### Differences of SHSE-S distribution between generation and validation dataset

The distribution of standardized score of social health were also listed in Table 1. Participants in the generation dataset had a slightly but significantly higher standardized score of social health comparing to the validation dataset. No significant differences were observed when we compared the number of participants between generation and validation datasets according to the decile of standardized score in the generation dataset using Chi-square goodness of fit test (*P* = 0.313) as Supplemental Table 3 showed.

### Regression-based normative data of SHSE-S for Zhejiang population

We combined generation and validation datasets to establish normative data of SHSE-S in Zhejiang elderly population. A regression-based norming approach was used to adjust for the influence of demographic factors. No existence of multicollinearity or influential values of the final models were detected. Homoscedasticity was also satisfied in all 4 final models. Table 2 showed that the predictors included in the final model to predict the standarized scores of SHSE-S and SS, SA, PER. Female, living in the rural community were positive predictors for SS while higher age, being widowed, divorced or never married and with prevalent chronic diseases were negative predictors. Participants who were female or with higher educational level tended to have a better status of SA. As for PER, female and higher education level were positive indicators while higher age, lived in the rural community and more prevalent chronic diseases were negative indicators. Results of Altman-Bland method showed that the proportions of predicted scores lying within acceptable range were 95.4, 95.1, 95.7, 95.3% for SHSE-S, SS, SA, and PER, respectively.

**Table 2:** Summary of the significant predictors included in the final regression models for SHSE-S scores

	Social Health Model		Social Support Model		Social Adjustment Model		Perceived Environment Resource Model	
Predictors	β (95%CI)	*P* value^a^	β (95%CI)	*P* value^a^	β (95%CI)	*P* value^a^	β (95%CI)	*P* value^a^
Intercept	41.20 (39.93, 42.48)	< 2 × 10^− 16^	19.57 (18.36, 20.79)	< 2 × 10^− 16^	45.09 (43.46, 46.71)	< 2 × 10^− 16^	51.06 (49.72, 52.39)	< 2 × 10^− 16^
Gender								
Male	0 (ref)	–	0 (ref)	–	0 (ref)		0 (ref)	
Female	2.43 (1.29, 3.57)	< 3 × 10^−5^	1.58 (0.02, 1.81)	0.003	5.26 (4.13, 6.39)	< 2 × 10^−16^	1.12 (0.34, 1.90)	0.005
Age	−0.14 (−0.32, 0.04)	0.140	0.19 (0.02, 0.23)	0.004	− 0.12 (− 0.23, − 0.01)	0.035	0.11 (0.02, 0.20)	0.003
Age^2^	−0.01 (− 0.02, − 0.006)	< 2 × 10^−5^	–	–	− 0.01 (− 0.02, − 0.01)	0.0002	−0.01 (− 0.02, − 0.01)	8 × 10^−6^
Education	0.72 (− 0.08, 1.52)	0.076	−0.31 (− 4.72, − 2.48)	1 × 10^− 6^	3.43 (2.70, 4.16)	< 2 × 10^− 16^	1.91 (0.73, 3.09)	0.002
Education^2^	− 0.75 (− 1.09, − 0.41)	< 2 × 10^− 5^	−1.81 (− 2.24, − 1.39)	5 × 10^− 15^	–	–	–	–
Marital status								
Married	0 (ref)		0 (ref)	–	0 (ref)		–	–
Widowed	−3.27 (− 4.34, − 2.20)	< 2 × 10^− 9^	− 6.20 (− 9.66, − 6.88)	0.0004	0.31 (− 1.37, 1.99)	0.717	–	–
Divorced, never married or other	−3.71 (− 4.98, − 2.43)	< 1 × 10^− 8^	− 4.22 (− 9.82, − 6.48)	0.207	−3.04 (− 5.10, − 0.99)	0.004	–	–
Living arrangement								
Living with others	0 (ref)		0 (ref)	–	–	–	0 (ref)	–
Living alone	−3.37 (− 5.76, − 0.98)	0.003	− 6.69 (− 6.80, − 1.93)	0.045	–	–	− 3.30 (− 5.43, − 1.17)	0.002
Region								
Urban	0 (ref)		0 (ref)	–	0 (ref)	–	0 (ref)	–
Rural	−4.58 (− 5.80, − 3.36)	< 2 × 10^− 13^	9.04 (6.59, 8.54)	6 × 10^−13^	−9.85 (− 11.08, − 8.62)	< 2 × 10^− 16^	−9.76 (− 11.12, − 8.39)	< 2 × 10^− 16^
Comorbidity								
0	–	–	0 (ref)		0 (ref)		0 (ref)	–
1	–	–	−1.87 (− 2.59, − 0.25)	0.013	−1.22 (− 2.75, 0.10)	0.068	− 0.80 (− 2.36, 0.76)	0.315
2	–	–	−1.96 (− 2.43, 0.37)	0.095	− 1.33 (− 2.82, 0.38)	0.135	−2.44 (− 5.02, 0.13)	0.062
3 or above	–	–	− 6.03 (− 3.03, − 9.16)	0.0001	−1.37 (− 3.01, − 0.27)	0.001	− 4.11 (− 7.51, − 0.71)	0.018
Interaction Items								
Age*Comorbidity	–	–	−0.09 (− 0.12, − 0.02)	0.001	–	–	− 0.08 (− 0.12, − 0.04)	0.0003
Region*Comorbidity	–	–	–	–	–	–	1.28 (0.57, 1.99)	0.0004
Model Performance								
Adjusted R^2^	0.36		0.36		0.39		0.36	
Cross-validation R^2^	0.35		0.33		0.37		0.35	
10-fold cross-validation R^2^	0.34		0.33		0.36		0.33	

^a^: Threshold for significance was 0.00625, 0.0056, 0.00625 and 0.0056 after Bonferroni correction in the Social Health model, Social Support model, Social Adjustment model and Perceived Environment Resource model, respectively

Normative data for SHSE-S and SS, SA, PER were established using a four-step approach as described above. For example, a participant who was female, 75 years old, married and now lived together with her husband in a rural community, middle school education level and self-claimed only had diabetes and hypertension with a 38 raw score of SHSE-S, her predicted score of SHSE-S would be 35.90. Then the residual should be calculated and standardized (standard residual = 0.297). And according to the standard normal cumulative distribution function, the percentile is 62 for this participant, which means about 62% of the normative population with similar demographic factors had a lower social health status comparing to this specific participant.

**Fig. 1:**
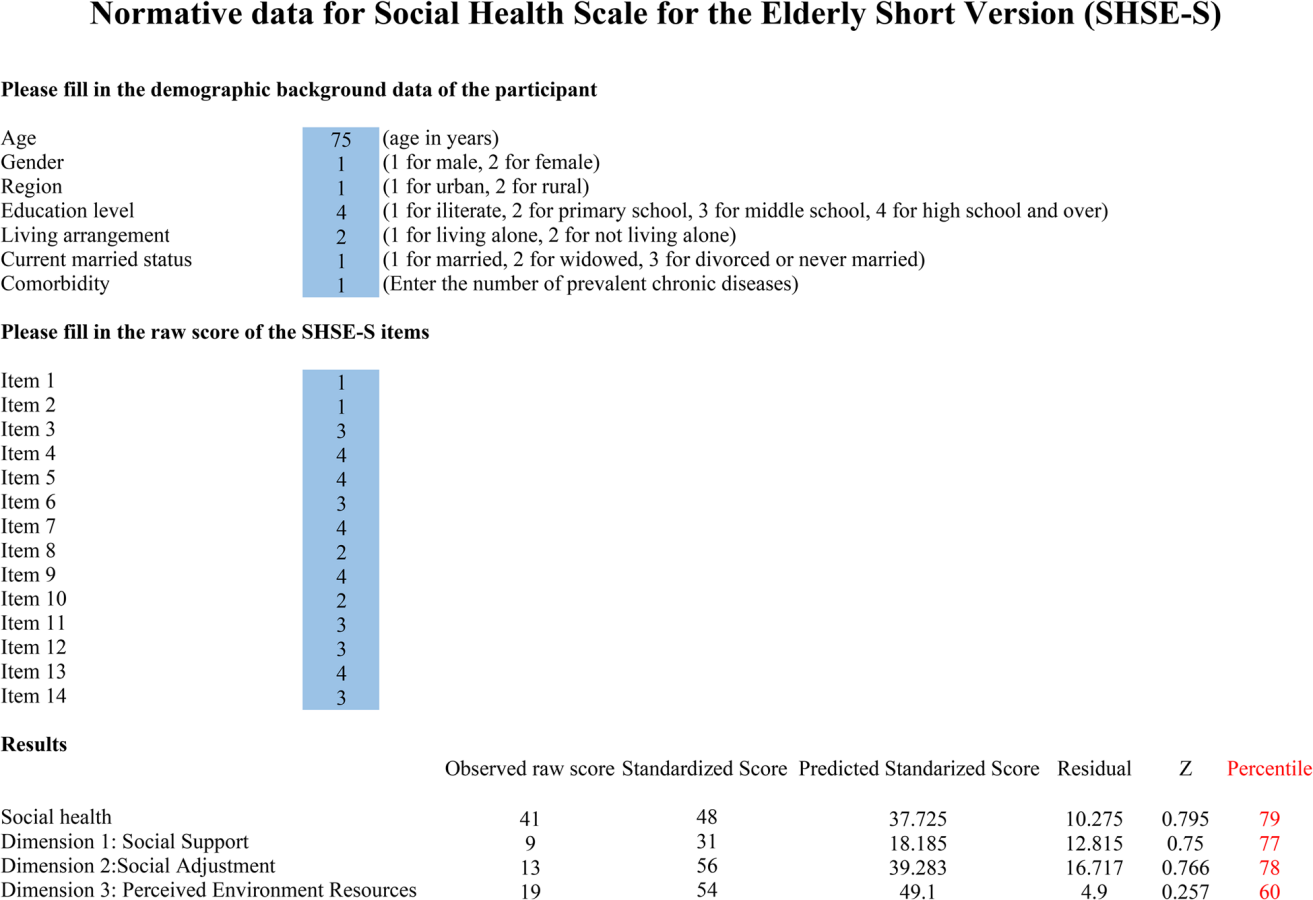
Screenshot of the SHSE-S excel scoring sheet

The four-step regression-based norming method provided accurate norms but the process was complicated since users had to repeat the computations to get the exact percentile. Therefore, simplified normative tables for standardized score stratified by region, gender and age which derived from regression norming procedure were provided in Table 3. Use of simplified tables of normative data is straightforward but lacks some accuracy because only a limited number of percentiles can be presented due to space and the age have to be rounded up. Thus, we built a computer-based algorithm in an Excel worksheet to maximize both accuracy and user-friendliness. A Screenshot of this worksheet was shown in Fig. 1. The users need to enter demographic factors and raw scores of all items of SHSE-S into the blue squares and the worksheet would automatically convert the scores into percentile using a four-step approach as described above. This worksheet can be derived from a reasonable request from the corresponding author.

**Table 3:** Normative data of SHSE-S standardized score for the elderly stratified by region, gender and age

Region	Percentile	Male& Female all ages	Male				Female			
	(%)		All ages	60–69	70–79	≥80	All ages	60–69	70–79	≥80
Rural	5	21	18	21	19	17	21	25	21	15
	10	26	27	27	27	19	27	29	25	19
	20	32	33	33	31	31	33	31	35	31
	30	38	37	37	35	33	37	37	38	35
	40	42	40	40	38	37	38	38	40	37
	50	45	42	42	41	38	44	44	42	42
	60	48	48	48	50	40	46	48	46	46
	70	51	50	52	52	44	50	50	50	48
	80	56	56	56	56	54	54	58	54	50
	90	62	62	63	62	56	60	62	62	54
	95	65	65	65	65	65	65	67	65	62
	99	77	75	75	73	71	77	79	77	79
Urban	5	27	27	29	27	23	27	29	29	21
	10	33	30	33	33	29	33	35	35	25
	20	38	37	37	37	35	38	40	40	33
	30	42	40	42	40	37	42	44	44	37
	40	46	44	44	42	38	46	50	50	42
	50	50	48	48	48	44	50	52	52	46
	60	54	52	52	52	48	54	56	56	50
	70	58	56	58	56	56	60	62	60	54
	80	61	60	62	62	58	62	63	63	56
	90	67	67	67	67	63	67	69	69	62
	95	73	71	73	73	69	75	75	75	63
	99	81	79	79	79	77	83	83	81	75

## Discussion

Results of the current study showed no heterogeneity was found between the distribution of SHSE-S between generation and validation datasets. We used a regression-based norming approach rather than traditional norming table which usually separated the sample by age and gender to build normative data for further research demands.

In this study we observed the mean scores of SHSE-S of generation dataset was higher than that in the validation dataset. This can be explained by the different distribution of demographic factors which had an impact on social health scales. In this study, we found significant demographic factors correlated with social health status. Multivariate linear regression showed that age, comorbidity (number of chronic diseases), living alone were negative indicators of social health while higher educational level, being female and being married. Female elderly had a significantly higher status of social health comparing to male elderly although the difference was modest. From the results of all item scores, we found that female elderly tended to perceive more social support and attend more social activities compared to male. These findings were consistent with previous researches [23, 24]. Another interesting disparity of social health status was found between urban and rural elderly population. Urban elderly possessed a higher social health score comparing to rural elderly. However, rural elderly had a higher level of social support while urban elderly had higher levels of social adjustment and perceived environment resources [25]. This can be explained that people lived in rural usually connected more closely with the neighborhood they lived in but the organized activities and community facilities were much better in the urban area [26, 27]. Although underlying the disparity of social health status is beyond the objective of the current study, these results could provide useful information for caring community-dwelling old adults. Further longitudinal researches should be conducted to explore the possible mechanism between demographic factors and social health status.

We built a regression-based norm for the elderly population in Zhejiang, China taking the effect of demographic factors into consideration. The regression-based approach mainly has two advantages comparing to the traditional strategy. First, it showed the researcher which variables were predictive of scale scores and therefore relevant to a more valid procedure compared with traditional procedure only considered age and gender. The second advantage is that norms are more continuous and more reliable than those obtained by tabulating the mean and standard deviation of the scores of different age*gender groups. Traditional procedure wasted a huge proportion of sample because it had to be split into different subgroups, while regression-based taken all covariates together into consideration and do not have the concern of losing sample size. Covariates in the regression-based approach didn’t need to round up (take age as an example, 69 had to be round up to 70 in the traditional way which undoubtfully lost some accuracy) thus the derived norms were much more smoothly. We suggest clinicians to use the current normative data to interpret the results of SHSE-S, but readers would have to refer to the norm of Bao et al. if they chose to use the SHSE-L (25 items).

The strength of the current study included the adoption of a regression-based norming approach which considers both accuracy and utility and relatively large sample size. However, there are several limitations that should be noted. First, the Cronbach’s α indexes for SA, PER were less than 0.7 (0.691 and 0.662, respectively) which showed that the internal consistency reliability was lower than the optimum level. And only construct validity and internal consistency reliability was tested in the current study, other indexes such as concurrent validity, test-retest reliability were examined in the sub-sample (participants from Gongshu and Xihu) and have been reported elsewhere [9]. The results showed that the reliability and validity of SHSE-S were acceptable but far from perfect. Second, although we found a significant relationship between comorbidity and social health status, the number of chronic diseases was based on self-report. There could be information bias that exist due to lack of medical knowledge, lack of access to medical service or other reasons. Third, China is a multi-ethnic society but the current study only included the Han population which restricts the generational utility of our scale. Considering the diversity and disparity of culture between different ethnicities in China, specific versions of SHES should be revised and developed in the future. Besides, other language versions of SHSE (such as Turkish version) is under preparing. Four, it should be noted that volunteer bias still exists due to the hard implementation of random sampling in the large-scale cross-sectional survey.

## Conclusion

In this study, data derived from a large sample in Zhejiang Province, China proved that SHSE-S was a useful tool for evaluating social health status for the elderly. Demographic factors such as age, gender, educational level were important predictors for social health status. Normative data and algorithms in the current study can be used as the reference for assessing and improving social health in the elderly population.

## Supplementary information

**Additional file 1: Table S1** Raw scoring strategy for each item in SHSE-S. **Table S2** Formulas for scoring and transforming for SHSE-S. **Table S3** Distribution of social health in generation and validation datasets according to regression-based norm.

## Data Availability

The datasets generated during and/or analyzed during the current study are not publicly available, but are available from the request to the corresponding author.

## Abbreviations

PER: Perceived environment resource
SA: Social adjustment
SHSE-S: Social health scale for the elderly short version
SS: Social support
