# Supplementary material for: Regression-based normative data for social health scale for the elderly (short version) in eastern China
Source: Health Qual Life Outcomes. 2020 Mar 4;18:54. doi: 10.1186/s12955-020-01306-2 (PMC7057499; doi:10.1186/s12955-020-01306-2)
Supplement: Supplementary file 1 — Additional file 1: Table S1 Raw scoring strategy for each item in SHSE-S. Table S2 Formulas for scoring and transforming for SHSE-S. Table S3 Distribution of social health in generation and validation datasets according to regression-based norm. [file 12955_2020_1306_MOESM1_ESM.docx]

| **Table S1** Raw scoring strategy for each item in SHSE-S | | | |
| --- | --- | --- | --- |
| Dimensions | Items | Response to Items | Scoring of raw score |
| Social Support | 1. Being supported in major decision | Nobody | 1 |
|  |  | 1 social roles | 2 |
|  |  | 2 social roles | 3 |
|  |  | 3 social roles | 4 |
|  |  | more than 3 social roles | 5 |
|  | 2. Being emotional cared for | Nobody | 1 |
|  |  | 1 social roles | 2 |
|  |  | 2 social roles | 3 |
|  |  | 3 social roles | 4 |
|  |  | more than 3 social roles | 5 |
|  | 3. Being comforted | Nobody | 1 |
|  |  | 1 social roles | 2 |
|  |  | 2 social roles | 3 |
|  |  | 3 social roles | 4 |
|  |  | more than 3 social roles | 5 |
|  | 4. Being helped with daily chores during illness | Nobody | 1 |
|  |  | 1 social roles | 2 |
|  |  | 2 social roles | 3 |
|  |  | 3 social roles | 4 |
|  |  | more than 3 social roles | 5 |
| Social Adjustment | 5. Participating in collective recreational activity | 1. less than 1 time per 3 months | 1 |
|  |  | 2. 1-2 times per 3 months | 2 |
|  |  | 3. 1-4 times per month | 3 |
|  |  | 4. 2-4 times per week | 4 |
|  |  | 5. more than 4 times per week | 5 |
|  | 6. Communication with children | 1. less than 1 time per 3 months | 1 |
|  |  | 2. 1-2 times per 3 months | 2 |
|  |  | 3. 1-4 times per month | 3 |
|  |  | 4. 2-4 times per week | 4 |
|  |  | 5. more than 4 times per week | 5 |
|  | 7. Communication with friends | 1. less than 1 time per 3 months | 1 |
|  |  | 2. 1-2 times per 3 months | 2 |
|  |  | 3. 1-4 times per month | 3 |
|  |  | 4. 2-4 times per week | 4 |
|  |  | 5. more than 4 times per week | 5 |
|  | 8. Spend leisure time on interests and hobbies | 1.Not even a little | 1 |
|  |  | 2. Only watch TV | 2 |
|  |  | 3. Minority of leisure time | 3 |
|  |  | 4. Half of leisure time | 4 |
|  |  | 5. Majority of leisure time | 5 |
| Perceived Environmental Resources | 9. Manufactured landscape in the community | 1. Not even a little | 1 |
|  |  | 2. 1 kind of landscape | 2 |
|  |  | 3. 2 kinds of landscape | 3 |
|  |  | 4. 3 kinds of landscape | 4 |
|  |  | 5. more than 3 kinds of landscape | 5 |
|  | 10. Time takes to walk to public transit facility | 1. No facility or more than 30 minutes | 1 |
|  |  | 2. 21-30 minutes | 2 |
|  |  | 3. 11-20 minutes | 3 |
|  |  | 4. 6-10 minutes | 4 |
|  |  | 5. ≤5 minutes | 5 |
|  | 11. Time takes to walk to fitness/recreation facility | 1. No facility or more than 30 minutes | 1 |
|  |  | 2. 21-30 minutes | 2 |
|  |  | 3. 11-20 minutes | 3 |
|  |  | 4. 6-10 minutes | 4 |
|  |  | 5. ≤5 minutes | 5 |
|  | 12. Time takes to walk to medical institution | 1. No facility or more than 30 minutes | 1 |
|  |  | 2. 21-30 minutes | 2 |
|  |  | 3. 11-20 minutes | 3 |
|  |  | 4. 6-10 minutes | 4 |
|  |  | 5. ≤5 minutes | 5 |
|  | 13. Frequency of organizing activity by local community | 1.Never | 1 |
|  |  | 2. 1-2 times per year | 2 |
|  |  | 3. 3-4 times per year | 3 |
|  |  | 4. 5-6 times per year | 4 |
|  |  | 5. More than 6 times per year | 5 |
|  | 14. Free public service provided by local community | 1. None | 1 |
|  |  | 2. 1-2 kinds of services | 2 |
|  |  | 3. 3-4 kinds of services | 3 |
|  |  | 4. 5-6 kinds of services | 4 |
|  |  | 5. more than 6 kinds of services | 5 |

**Table S2** Formulas for scoring and transforming for SHSE-S

| Dimension | Sum final item values | Lowest and highest possible raw scores | Possible raw score range |
| --- | --- | --- | --- |
| Social support | 1+2+3+4 | 4, 20 | 16 |
| Social adjustment | 5+6+7+8 | 4, 20 | 16 |
| Perceived environment resources | 9+10+11+12+13+14 | 6, 30 | 24 |

**Table S3** Distribution of social health in generation and validation datasets according to regression-based norm

| Decile of the norm | Generation dataset (N=2392) | Validation dataset (N=3697) |
| --- | --- | --- |
| 1 | 240 (10.03%) | 386 (10.44%) |
| 2 | 225 (9.41%) | 374 (10.12%) |
| 3 | 217 (9.07%) | 346 (9.36%) |
| 4 | 257 (10.74%) | 427 (11.55%) |
| 5 | 207 (8.65%) | 347 (9.39%) |
| 6 | 257 (10.74%) | 414 (11.20%) |
| 7 | 230 (9.62%) | 360 (9.74%) |
| 8 | 273 (11.41%) | 355 (9.60%) |
| 9 | 244 (10.20%) | 329 (8.90%) |
| 10 | 240 (10.03%) | 359 (9.71%) |

*P* value = 0.313 by Chi-square goodness of fit test.
